# SQI Quality Evaluation Mechanism of Single-Lead ECG Signal Based on Simple Heuristic Fusion and Fuzzy Comprehensive Evaluation

**DOI:** 10.3389/fphys.2018.00727

**Published:** 2018-06-14

**Authors:** Zhidong Zhao, Yefei Zhang

**Affiliations:** ^1^Hangdian Smart City Research Center of Zhejiang Province, Hangzhou Dianzi University, Hangzhou, China; ^2^School of Communication Engineering, Hangzhou Dianzi University, Hangzhou, China

**Keywords:** electrocardiogram(ECG), quality assessment, signal quality indexes (SQIs), heuristic fusion, fuzzy comprehensive evaluation

## Abstract

For both the acquisition of mobile electrocardiogram (ECG) devices and early warning and diagnosis of clinical work, high-quality ECG signals is particularly important. We describe an effective system which could be deployed as a stand-alone signal quality assessment algorithm for vetting the quality of ECG signals. The proposed ECG quality assessment method is based on the simple heuristic fusion and fuzzy comprehensive evaluation of the SQIs. This method includes two modules, i.e., the quantification and extraction of Signal Quality Indexes (SQIs) for different features, intelligent assessment and classification. First, simple heuristic fusion is executed to extract SQIs and determine the following SQIs: R peak detection match qSQI, QRS wave power spectrum distribution pSQI, kurtosis kSQI, and baseline relative power basSQI. Then, combined with Cauchy distribution, rectangular distribution and trapezoidal distribution, the membership function of SQIs was quantified, and the fuzzy vector was established. The bounded operator was selected for fuzzy synthesis, and the weighted membership function was used to perform the assessment and classification. The performance of the proposed method was tested on the database from Physionet ECG database, with an accuracy (Acc) of 97.67%, sensitivity (Se) of 96.33% and specificity (Sp) of 98.33% on the training set. Testing against the test datasets resulted in scores of 94.67, 90.33, and 93.00%, respectively. There's no gold standard exists for determining the quality of ECGs. However, the proposed algorithm discriminates between high- and poor-quality ECGs, which could aid in ECG acquisition for mobile ECG devices, early clinical diagnosis and early warning.

## Introduction

With the wide application of mobile ECG in the fields of financial safety, security monitoring, medical insurance, and data confidentiality, ECG recording devices are not limited to professional training staff.

From the perspective of mobile ECG collection, most of the available ECG recording devices lack real-time feedback about the signal quality. Thus, it is difficult for non-professionals to collect high-quality ECG signals. The mobile recorders record the ECG in normal lifestyle condition. So there is movement of the recorders electrodes (Clifford et al., 2006). The existence of noise prevents the accurate detection of important clinical characteristics, thus reducing the quality of the ECG signal (Tob'on and Falk, 2015).

Regarding the clinical application of ECG signals, an ECG signal contains abundant physiological and pathological information, which can help clinical staff observe the early warnings of diseases and make a diagnosis. For instance, for the diagnosis of cardiovascular disease (World Health Organization, 2016), the high costs of primary health care make follow-up treatment unaffordable. To circumvent this problem, many countries transmit real-time ECG data recorded by patients to clinical experts to diagnose patients. In addition to the professional judgment of clinical experts, the accuracy of remote diagnosis should reduce the number of low-quality ECGs sent to experts, so we need to determine whether the quality of recorded ECGs is sufficient. In addition, in the ICU early warning system, the high false positive rate of monitors is caused by noise and data loss (Lawless, 1994). A survey demonstrated that only 10% of the alerts are related to treatment (Allen and Murray, 1996; Chambrin et al., 1999), which increases the workload of ICU staff and ultimately desensitize them.

Therefore, establishing a suitable assessment mechanism that divides the signal results into several different levels is particularly important.

The technology for signal quality assessment is gradually emerging. Currently, numerous research reports regarding quality assessment technology for ECG signals are available. However, the gold standard of ECG quality has not been evaluated to date. According to the existing research results, the research ideas for evaluation methods of ECG signal quality can be roughly divided into the following four methods: the waveform shape of the time domain signal, the characteristics of each frequency band of the frequency domain signal, signal quality characteristics extracted using the nonlinear tool, and signal quality parameters.

In this paper, we discuss the correlation between ECG signal quality and noise and ECG waveform characteristics to obtain accurate assessment results using simple rules and complex classification techniques. Our method of assessing ECG quality is divided into two steps:

### Step1: feature extraction

Based on the noise characteristics and ECG waveform features, six quality assessment parameters are extracted and quantified: the matching degree of R peak detection, power spectrum distribution of QRS wave, variability in the R-R interval, kurtosis, skewness, and baseline relative power. The advantages and disadvantages as well as the accuracy, sensitivity, and specificity of each of the quality assessment parameters of ECG quality are obtained by conducting quality assessment on the six quality assessment parameters. Using a simple heuristic fusion operation, the best accuracy based on the combination of 2–6 parameters is selected.

### Step2: intelligent classification

Using the fuzzy comprehensive evaluation method that combines Cauchy distribution, rectangular distribution, and trapezoidal distribution, the membership of the signal to be evaluated is calculated based on the parameters of the logical combination of the best accuracy selected in step 1. By establishing the fuzzy vector and choosing bounded calculation Sub-fuzzy synthesis, the ECG signal is divided into the evaluation level set *V* = {*E, B, U*} through the principle of weighted membership decision-making division.

The above algorithm evaluation is based on a single-lead ECG signal. If ECG signals are collected for multiple leads that are independent of each other, each channel can be processed separately.

## Methods and materials

### Databases

To determine the parameters of the fuzzy comprehensive evaluation (correlation matrix R, weight vector A, synthesis operator) and verify the effectiveness of our algorithm.In this work, the ECG signals were obtained from ECG database (Physionet ECG database). We adopt two of these databases, Physionet/Cinc Challenge 2017 (Physionet, 2017), marked as database D1 and Physionet/Cinc Challenge 2011 (Physionet, 2011), marked as database D2.

The database D1 is single-lead ECG records, which is coincided with the ever-evolving mobile measurement and wearable measurement methods, supplied by AliverCor, which was annotated by clinical experts and categorized into one of four groups (i.e., normal rhythm, atrial fibrillation, other rhythm, and noisy recordings). We randomly selected 150 groups of normal rhythm, 150 groups of noisy recordings, and the length of each group is 9,000 data points.

The database D2 is standard 12-lead ECG recordings, in which the quality of ECGs were reviewed and examined by a group of annotators with varying amounts of expertise in ECG analysis. In our present work, we have used only lead II(Set-a) and randomly selected 150 groups of acceptable, 150 groups of unacceptable, and the length of each group is 9,000 data points.

To maximize contrast and create a balanced database, a 10-fold cross-validation method was used 10 times to reduce the generalization error in the training set (Zhihua, 2016), Figure 1 below shows the schematic diagram of 10-fold cross validation.

**Figure 1:**
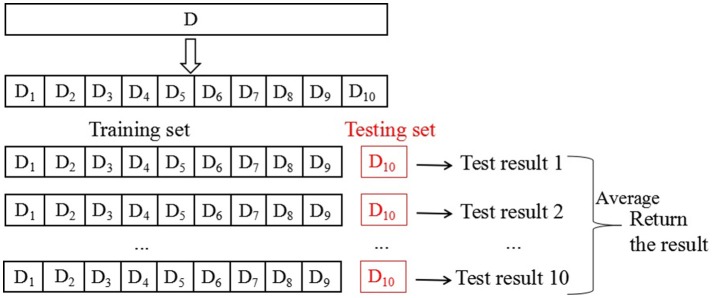
Schematic of 10-fold cross-validation method repeated 10 times.

The database is first divided into 10 equally sized mutually exclusive subsets: *D* = *D*_1_∪*D*_2_∪…∪*D*_10_. *D*_*i*_∩*D*_*j*_ is empty. Each subsection maintains the consistency of the data distribution, which is obtained through hierarchical sampling from D. Then, every nine subsets of the union is considered as a training set, and the remaining subset serves as the test set. Thus, 10 training and test sets are obtained. Thus, we can conduct 10 training and testing assessments and obtained the mean of 10 final test results. In this paper, the mean of 10 test sets is used as the result of cross validation to evaluate the performance of the algorithm. Numerous methods are available to divide database D into 10 subsets. To reduce the difference caused by different sample divisions, we randomly apply different division methods by repeating the process 10 times. The resulting assessment is the average of the 10 replicates of the 10-fold cross-validation results, the mean of the obtained results is the final performance indicator. Obviously, the stability and fidelity of the cross-validation method evaluation results are considerably improved compared with those of the commonly used single-division leave-one-out method.

### Method outline

**Figure 2:**
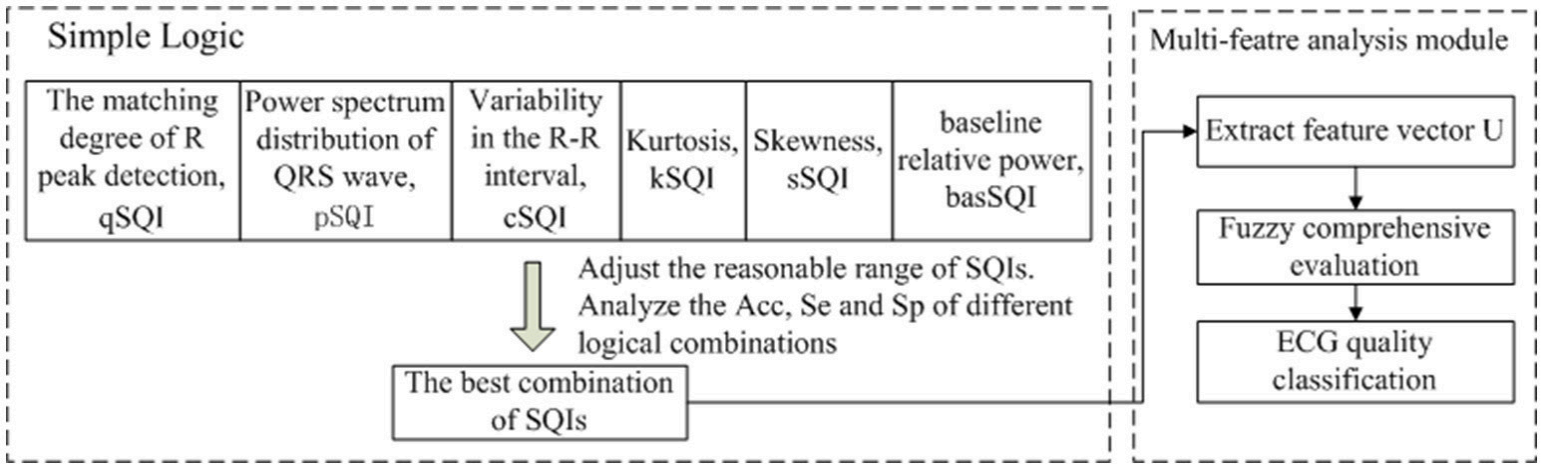
The flowchart of the proposed method, which includes two modules.

Two methods for fusing the signal quality information were compared. The first step is based on simple heuristic fusion. Through the ECG waveform characteristics, time-frequency characteristics, and the time-frequency characteristics of the noise source, we propose six quality evaluation parameters and adjust the reasonable range of SQI. ECG quality [accuracy (Acc), sensitivity (Se) and specificity (Sp)] was analyzed using a simple logical combination to obtain the best combination of signal quality indexes (SQIs) *U* = {*u*_1_, *u*_2_, *u*_3_, …}. The second step applied fuzzy comprehensive evaluation, which represents a more accurate assessment and classification of *U* = {*u*_1_, *u*_2_, *u*_3_, …}. A schematic representation of the proposed method is presented in Figure 2.

### Signal quality indexes (SQIs)

#### Matching degree of R peak detection qSQI

**Figure 3:**
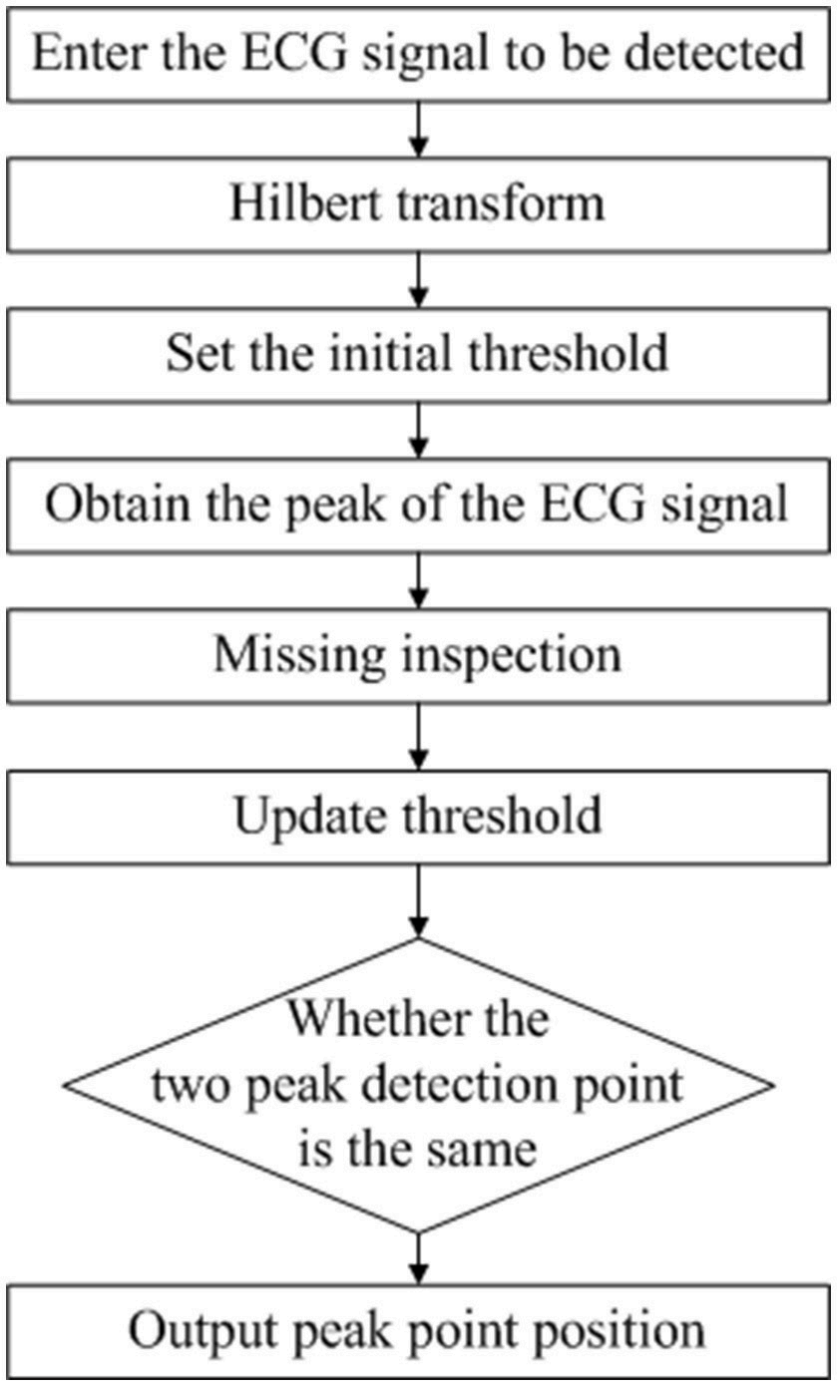
R Wave Detection Flow Chart Based on Hilbert and Dynamic Adaptive Threshold.

We next describe an evaluation index for ECG signal recognition ability. For a complete ECG signal, the R wave has the maximum amplitude and most obvious characteristic, so the existence of the ECG signal is often identified by R-wave detection. Therefore, using different algorithms to perform R-wave detection on the same ECG signal, the result is compared and analyzed to estimate the quality of the signal. In this paper, Hilbert and dynamic adaptive threshold based on R-wave detection (algorithm flow shown in Figure 3 and denoted as Algorithm 1), wavelet transform (This process is denoted as Algorithm 2) are used to compare the same ECG signal detection results. As for wavelet transform, the input ECG signal was decomposed by discrete wavelet transform (DWT) with four layers, and the wavelet coefficients for each layer were obtained. The R point is the singularity of the ECG waveform. The extraction of the R point is completed by the correspondence between the singularity of the signal and the positive and negative modulus maximum of the wavelet coefficients to the zero point (Zhen et al., 2008).

Any R-wave detection algorithm has certain shortcomings, which may lead to false positives. Accordingly, the ECG signal quality is evaluated by the same R-wave matching degree of the two algorithms mentioned above. The following equation is used to obtain the QRS wave R peak detection matching degree:

(1) $$qSQI=\frac{2N}{N_{a}+N_{b}}$$

where *N* indicates the correct number of R waves detected by the two algorithms and *N*_*a*_ and *N*_*b*_ denote the numbers of R waves measured by Algorithms 1 and 2, respectively. The identification criteria of qSQI are given as follows:

(2) $$ECG\;\{\begin{array}{ll} optimal, & qSQI>90\%;\\ suspicious, & qSQI\in \left[60\%,\;90\%\right];\\ \text{unqualified}, & qSQI<60\% \end{array}$$

#### Power spectrum distribution of QRS wave pSQI

We next describe an evaluation index of QRS wave quality (Li and Clifford, 2006). A heartbeat cycle is mainly composed of a P wave, QRS complex wave, T wave, and other important eigenvectors, of which the QRS wave accumulates ~99% of the energy of the ECG signal and is the most stable. The energy of the QRS wave is concentrated in a frequency band centered at 10 Hz and is 10 Hz in width. Therefore, pSQI is mathematically defined as follows:

(3) $$pSQI=\frac{\int_{f=5Hz}^{f=15Hz}P\left(f\right)df}{\int_{f=5Hz}^{f=40Hz}P\left(f\right)df}$$

Spectrum analysis is performed, and the energy of the two bands is calculated. The numerator represents the energy of the QRS wave, and the denominator represents the overall energy of the ECG signal.

If EMG interference exists, the high-frequency component increases, and pSQI decreases. The identification criteria of pSQI are given as follows:

(4) $$ECG\;\{\begin{array}{l} optimal\;pSQI\in [l_{1},l_{2}];\\ suspicious,\;pSQI\in [l_{3},l_{1}];\\ unqualified\;pSQI>l_{2},or\;pSQI<l_{3}; \end{array}$$

*l*_1_ and *l*_2_, which represent the lower and upper limits, respectively, vary slightly with the heart rate. Based on the experimental training set, we adjusted *l*_1_ and *l*_2_ as follows:

(5) $$\text{Heart rate}\{\begin{array}{l} \in [\text{60bmp},\text{ 130bmp}],l_{1}=0.5,l_{2}=0.8;l_{3}=0.4;\\ \in [\text{130bmp},\text{160bmp}],l_{1}=0.4,l_{2}=0.7;l_{3}=0.3; \end{array}$$

#### Variability in the R-R interval cSQI

We next describe an evaluation index of normal and stable heart rhythm. The ECG signal is a periodic signal, and the interval of R-R interval is periodic. The calculation of cardiac cycle (single-cycle ECG length) is related to the heart rate, which differs depending on the exercise state. The measurement is very sensitive to motion artifacts in the human body; the ECG signal is collected in the active state. We need to ensure that the heart rhythm has a normal ECG signal to avoid affecting the clinical diagnosis. In addition, the presence of noise artifacts leads to reduced QRS detector performance. When an artifact is present, the QRS detector underperforms by either missing R-peaks or erroneously identifying noisy peaks as R-peaks. The above two problems will lead to a high degree of variability in the distribution of R-R intervals; therefore, the coefficient of the variation of R-R interval proposed by Hayn (Hayn et al., 2012) was used to calculate the variability of R-R intervals:

(6) $$cSQI=\frac{{\hat{\sigma }}_{RR}}{{\hat{\mu }}_{RR}}$$

where ${\hat{\mu }}_{RR}$ and ${\hat{\sigma }}_{RR}$ are the empirical estimates of the mean and standard deviation of the distribution of the R-R intervals within a segment of ECG. The identification criteria of cSQI are given as Equation (7), where the threshold was determined empirically.

(7) $$ECG\;\{\begin{array}{l} optimal\;cSQI<0.45;\\ suspicious\;qSQI\in \left[0.45,\;0.64\right];\\ \text{unqualified},\;qSQI>0.64 \end{array}$$

#### Skewness sSQI and kurtosis kSQI

We next describe an evaluation index of the de-noising effect of three disturbing noises, which is defined as follows:

(8) $$sSQI=\nu_{3}=\frac{E\left\{{\left(x-\mu_{x}\right)}^{3}\right\}}{\sigma^{3}}$$

(9) $$kSQI=\nu_{4}=\frac{E\left\{{\left(x-\mu_{x}\right)}^{4}\right\}}{\sigma^{4}}$$

The third and fourth standardized moments of a signal are measures of signal symmetry and Gaussianity, respectively. The central limit theorem indicates that random uncorrelated processes tend to have Gaussian distributions, such as thermal noise. Conversely, correlated signals tend to exhibit non-Gaussian distributions. The fourth standardized moment of a distribution, kurtosis, measures the relative peakedness of a distribution with respect to a Gaussian distribution. However, outliers will cause the asymmetric distribution of the signal, and the skewness is high. In this equation, μ_*x*_ and σ are the mean and standard deviation of the signal, respectively.

**Figure 4:**
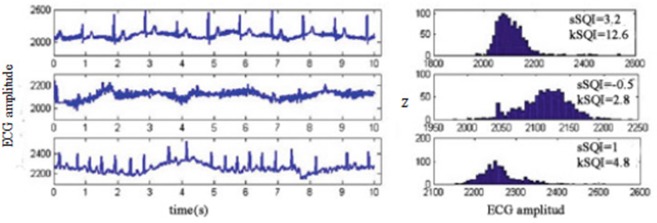
Skewness-kurtosis distribution for different quality ECG signals.

Figure 4 shows different quality ECG signals and their kurtosis and skewness values. Comparing Example 1 with the other three sets of signals, the noisy ECG signal has a smaller kurtosis value but a different skewness distribution. Example 2 contains a large amount of high-frequency noise. The kurtosis value is very low. However, given the nature of the noise, the distribution is approximately symmetric, yielding a low skewness value. Therefore, skewness is less robust to noise than kurtosis, and we only use kurtosis in the ensuing noise detection. The kSQI in each case is described as follows:

1. For a standard, noise-free and normal sinus ECG, the value is >5 (He et al., 2006);
2. If power frequency interference, baseline drift or Gaussian distribution of random noise is noted, the values is < 5 (Clifford et al., 2006);
3. If EMG interference is present, the value is ~5 (He et al., 2006);

The identification criteria of kSQI is given as follows:

(10) $$ECG\;\{\begin{array}{l} optimal\;kSQI>5;\\ unqualified,\;kSQI\leq 5; \end{array}$$

#### The relative power in the baseline basSQI

**Figure 5:**
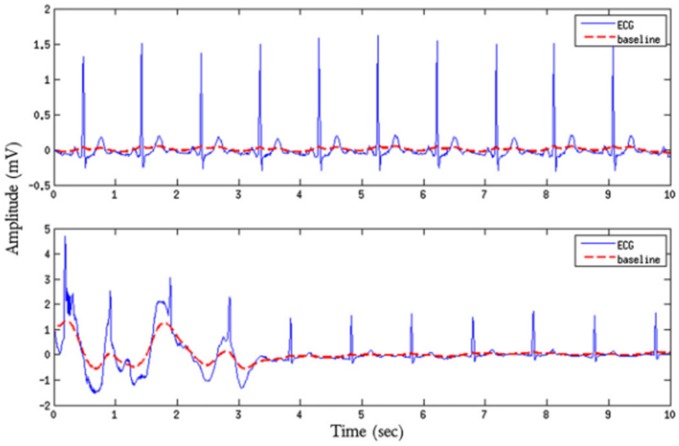
ECG quality comparison chart with or without baseline shift.

We next describe an evaluation index of the de-noising effect of baseline drift (Zhihua, 2016). basSQI is difficult to filter, but its presence greatly impacts late pathological judgment and identification, as shown in Figure 5, which gives an example of baseline for a high-quality ECG sample (upper plot, *basSQI* = 0.966) and low-quality ECG sample (lower plot, *basSQI* = 0.5) obtained from Set-a of the PhysioNet/CinC 2011 database (Silva et al., 2011). Therefore, it is necessary to evaluate its de-noising effect, as defined below:

(11) $$basSQI=\frac{1-\int_{f\;=\;0Hz}^{f\;=\;1Hz}P\left(f\right)df}{\int_{f\;=\;0Hz}^{f\;=\;40Hz}P\left(f\right)df}$$

If no baseline drift interference is noted, the basSQI value is close to 1. A low basSQI means that the power within the band [0, 1*Hz*] is abnormally high with respect to the power in the [0, 40*Hz*] interval, which is likely to be caused by an abnormal shift in the baseline. The identification criteria of basSQI are given as follows:

(12) $$ECG\;\{\begin{array}{ll} optimal, & basSQI\in [0.95,\;1];\\ suspicious, & basSQI\in [0.9,\;0.95];\\ unqualified, & basSQI<0.9; \end{array}$$

### Data fusion approaches

#### Simple heuristic fusion of the SQIs

After the analysis in the previous section, five SQIs are reserved. The simple logic classifier model was built on database D, and the performance of the individual SQIs was evaluated. Then, we studied the contribution of each SQI to the classification performance and removed SQIs with an accuracy (Acc) < 75%. Then, we evaluated of all other possible combinations of SQIs (pairs, triplets, etc.). Ten-fold cross-validation was performed on database D to assess the performance of the predictive model. Possible fusion equations were constructed in an ad hoc manner as follows:

(13) $$\begin{array}{l} \left({\text{SQI}}_{\text{1}},{\text{SQI}}_{\text{2}}\right):ECG\;is\\ \;\{\begin{array}{ll} Excellent(E), & \prime optimal\prime =2;\\ \text{Barely acceptable}(B), & \prime suspicious\prime =2\;or\\ \; & \prime optimal\prime =1,\prime suspicious\prime =1;\\ Unacceptable(U), & others; \end{array} \end{array}$$

(14) $$\begin{array}{l} \left(SQI_{1},SQI_{2},SQI_{3}\right):ECG\;is\\ \;\{\begin{array}{ll} Excellent(E), & \prime optimal\prime \geq 2,\prime unqualified\prime =0;\\ \text{Barely acceptable}(B), & others;\\ Unacceptable(U), & \prime unqualified\prime =2\;or\\ \; & \prime suspicious^{\prime }=2,\prime unqualified\prime =1; \end{array} \end{array}$$

(15) $$\begin{array}{l} \left({\text{SQI}}_{\text{1}},{\text{SQI}}_{\text{2}},{\text{SQI}}_{\text{3}},{\text{SQI}}_{\text{4}}\right):ECG\;is\\ \;\{\begin{array}{ll} Excellent(E), & \prime optimal\prime \geq 3,\prime unqualified\prime =0;\\ \text{Barely acceptable}(B), & others;\\ Unacceptable(U), & \prime unqualified\prime \geq 3\;or\\ \; & \begin{array}{l} \prime unqualified\prime =2,\prime suspicious^{\prime }\geq 1\;or\\ \prime unqualified\prime =1,\prime suspicious^{\prime }=3; \end{array} \end{array} \end{array}$$

(16) $$\begin{array}{l} \left({\text{SQI}}_{\text{1}},{\text{SQI}}_{\text{2}},{\text{SQI}}_{\text{3}},{\text{SQI}}_{\text{4}},{\text{SQI}}_{\text{5}}\right):ECG\;is\\ \;\{\begin{array}{ll} Excellent(E), & \prime optimal\prime \geq 4,\prime unqualified\prime =0;\\ \text{Barely acceptable}(B), & others;\\ Unacceptable(U), & \prime unqualified\prime \geq 4\;or\\ \; & \begin{array}{l} \prime unqualified\prime =3,\prime suspicious^{\prime }\geq 1\;or\\ \prime unqualified\prime =2,\prime suspicious^{\prime }\geq 2\;or\\ \prime unqualified\prime =1,\prime suspicious\prime =4; \end{array} \end{array} \end{array}$$

Where the ECG quality corresponding to the number distribution of “optimal,” “suspicious,” and “unqualified” is arbitrary and set empirically through trial and error. Although these coefficients could be optimized, it is unlikely that the logic is optimal. Thus, an exhaustive search of possible logical combinations and thresholds was not performed.

Multiple SQI metrics quantify different characteristics, and the simple fusion of the SQIs classifies the signal quality of each ECG into three levels: excellent (E), barely acceptable (B), and unacceptable (U). We obtained the best combination of quality assessment parameters *U* = {*u*_1_, *u*_2_, *u*_3_, …} by comparing the accuracy(Acc), sensitivity (Se) and specificity (Sp) of the different logical combinations. The three indicators are defined as follows:

(17) Se= TP/(TP+FN)

(18) Sp=TN/(TN+FP)

(19) Acc= (TN+TP)/(TP+TN+FN+FP)

TP (true case) indicates the number of acceptable ECG signals correctly counted. TN (true negative example) indicates the number of unacceptable ECG signals correctly counted. FP (false positives) indicates the number of acceptable ECGs under error statistics. FN (false positives) indicates the number of unacceptable ECGs that were counted as errors.

Table 1 shows the performance of five SQIs in ECG quality assessment. The table clearly shoes that qSQI and pSQI best distinguish between records of good and bad quality (the results obtained from the 300 sets in database D1 and D2). Then, the system is trained with all possible combinations of SQIs, using 10-fold cross-validation method repeated 10 times to train database D1 and D2, and merged with the upper (13)–(16). The results for the best pair, triplet, etc. of SQIs combinations are summarized in Table 2 and Table 3. Analysis of the table clearly reveals that as SQIs increase, the accuracy rate exhibited a slowly increasing trend. As for database D1, the best accuracy was obtained when considering all SQIs ((*Acc*)_*D*1_ = 87.00%). However, compared with 4 SQIs (with higher sensitivity, (*S*_*e*_4_)*D*1_ = 94.67% is superior to (*S*_*e*_5_)*D*1_ = 87.33%), the accuracy difference is negligible. As for database D2, it shows the best precision when only considering qSQI, pSQI, kSQI and basSQI. Accordingly, *U* = {*u*_1_, *u*_2_, *u*_3_, *u*_4_} = {*qSQI, pSQI, kSQI, basSQI*} is the best combination for evaluation factor aggregation in the fuzzy comprehensive evaluation mechanism.

**Table 1:** Single-lead classification using individual SQIs.

		**qSQI**	**pSQI**	**cSQI**	**kSQI**	**basSQI**
Database D1	Acc	**80.33**	**80.00**	76.00	**79.67**	**78.67**
	Se	95.33	95.00	63.67	84.33	80.67
	Sp	88.33	80.33	56.33	83.00	72.33
Database D2	Acc	**86.33**	**77.00**	74.33	**82.33**	**83.00**
	Se	93.67	84.33	66.33	85.00	86.00
	Sp	80.67	69.67	47.67	80.67	79.67

*The best performing SQI indicator is shown in bold and underlined*.

**Table 2:** Single-lead classification using combination of SQIs.

**SQI entered**	**Acc Training performance (%)**	**Acc Test performance (%)**
qSQI, pSQI	81.67	77.33
qSQI, pSQI, kSQI	83.33	81.00
qSQI, pSQI, kSQI, basSQI	**85.67**	**84.33**
qSQI, pSQI, kSQI, basSQI, cSQI	88.67	87.00

*The results is performed on Database D1. The best result of SQIs combinations (Database D1) is shown in bold and underlined*.

**Table 3:** Single-lead classification using combination of SQIs.

**SQI entered**	**Acc Training performance (%)**	**Acc Test performance (%)**
qSQI, pSQI	87.33	83.67
qSQI, pSQI, kSQI	88.67	87.00
qSQI, pSQI, kSQI, basSQI	**92.00**	**91.33**
qSQI, pSQI, kSQI, basSQI, cSQI	91.67	89.67

*The results is performed on Database D2. The best result of SQIs combinations (Database D2) is shown in bold and underlined*.

#### SQI quality evaluation mechanism based on fuzzy comprehensive evaluation

We selected the best SQIs combination in simple heuristic fusion, and the SQI quality evaluation mechanism based on fuzzy comprehensive evaluation was established. Fuzzy comprehensive evaluation is based on fuzzy mathematics by applying the principle of fuzzy relational synthesis, quantifying some undefined and unquantifiable factors, and using a number of factors to evaluate the affair level of a comprehensive evaluation of a method (Fengbiao, 2000). The fuzzy data fusion technology has mature applications in speech analysis (Song et al., 2016), image analysis (Wenqing and Yongjun, 2016), traffic network, and power grid risk assessment (Deng et al., 2017). The specific steps are as follows:

**First, determine the evaluation factor aggregation** *U*

For the evaluated object, select the main factors that reflect the evaluation object, measure with corresponding index, and form the evaluation factor aggregation *U*. The ECG signal's evaluation factor aggregation is *U* = {*u*_1_, *u*_2_, *u*_3_, *u*_4_} = {*qSQI, pSQI, kSQI, basSQI*}.

**Second, determine the rating hierarchy** *V*

For each evaluation factor, determine a number of levels. In this paper, the quality of ECG signal is divided into excellent (E), barely acceptable (B), and unacceptable (U). The evaluation rating set is *V* = {*v*_1_, *v*_2_, *v*_3_}.

**Then, establish the evaluation matrix** *R*

Analyze the membership function *r*_*ij*_ of each factor *u*_*i*_ to the rating level *v*_*j*_, and obtain the single factor evaluation result of the ith factor: *r*_*i*_ = (*r*_*i*1_, *r*_*i*2_, *r*_*i*3_). After the multiple single factor evaluation, a fuzzy matrix R is formed. In this paper, fuzzy matrix R, which has 4 factors and 3 evaluation levels, is described as follows:

$$R=\left[\begin{array}{ccc} r_{11} & r_{12} & r_{13}\\ r_{21} & r_{22} & r_{23}\\ r_{31} & r_{32} & r_{33}\\ r_{41} & r_{42} & r_{43} \end{array}\right]$$

**Next, determine the weight vector** *W*

According to the various factors for the evaluation of the importance of the object, give the appropriate weight, which is marked as *W* = (*w*_1_, *w*_2_, *w*_3_, *w*_4_), ∑*w*_*i*_ = 1. The W in this paper is based on 100 sets of experimental training to adjust the experimental training set.

**Then, assess fuzzy synthesis S**

*S* = *W*°*R*, *S* = (*s*_1_, *s*_2_, *s*_3_). Different operator symbols ° correspond to different fuzzy comprehensive evaluation models (Zimmermann, 2011).

**Finally, make a decision**

According to the assessment needs of the appraisers to process *S*, obtain the results.

**Figure 6:**
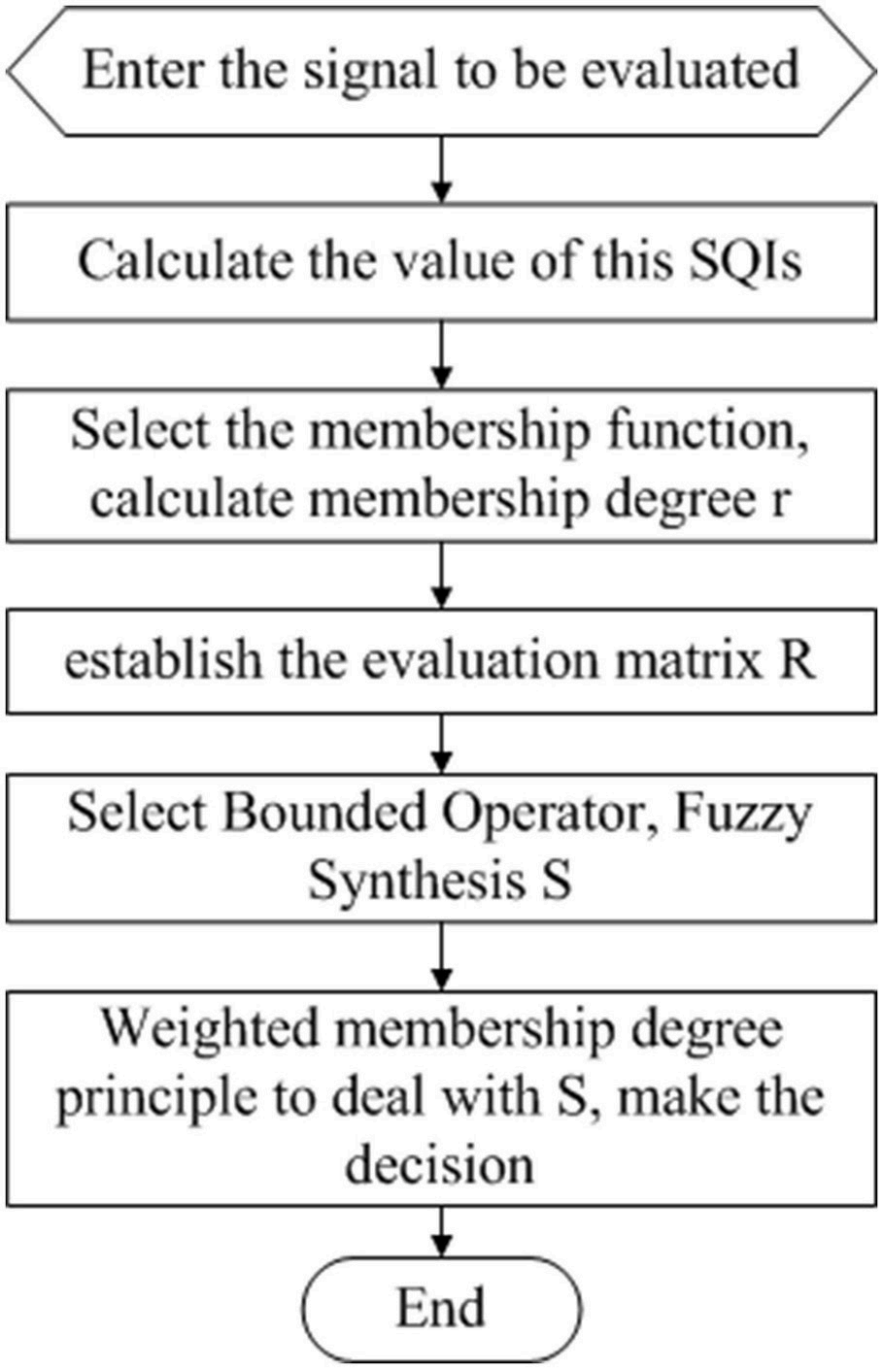
Single-factor evaluation flow chart.

The single factor evaluation process for each factor *u*_*i*_ is shown in Figure 6 below.

Therefore, with a rating hierarchy V, the key step of single factor evaluation is to calculate the membership function *r*_*ij*_ and determine the fuzzy operator symbols °. After determining the single factor evaluation, the key step in multifactorial evaluation is the choice of weight vector *W* and decision-making methods.

##### Determine the membership function of each single factor evaluation

*Single factor evaluation of qsqi*. Assuming that q is the value of matching degree of R peak detection, *q*∈[0, 100], we construct the membership function of its quality level (E, B, and U) as *U*_*qH*_(*q*), *U*_*qI*_(*q*), and *U*_*qJ*_(*q*), respectively. Due to the matching degree of R peak detection's performance level and because its corresponding evaluation object values approximate a Cauchy distribution, we choose the Cauchy distribution function to serve as the membership function of qSQI.

(a).*U*_*qH*_(*q*)

Based on the understanding of formula (1), the greater the value of q is, the greater the membership of H is. Therefore, we select the increasing half of the Cauchy distribution.

(20) $$U_{qH}\left(q\right)=\{\begin{array}{ll} 0, & q\leq a\\ \frac{1}{\left\{1+{\left[\alpha \left(q-a\right)\right]}^{-\beta }\right\}}, & q>a \end{array}$$

Specifically, α, β>0. In practice, we often utilize β = 2.

Analysis Equation (20), take *a* = 80. If q is not >80, the membership function to H is zero. If q is > 80, there is a non-zero membership for H. The greater the q value is, the greater the membership for H is. When *q* = 100, *U*_*qH*_(*q*) = 1, thus improving Equation (20):

(21) $$U_{qH}\left(q\right)=\{\begin{array}{ll} 0, & 0\leq q\leq 80\\ \frac{1}{\left\{1+{\left[\alpha \left(q-80\right)\right]}^{-\beta }\right\}}, & 80<q<90\\ \frac{x}{100}, & 90\leq q\leq 100 \end{array}$$

In addition, to ensure the continuity of *U*_*qH*_(*q*) to calculation a, $\underset{x\to 90^{-}}{\lim}\frac{1}{\left\{1+{\left[\alpha \left(q-80\right)\right]}^{-2}\right\}}=0.9$, yielding α = 0.3. Therefore, *U*_*qH*_(*q*) is as follows:

(22) $$U_{qH}\left(q\right)=\{\begin{array}{ll} 0, & 0\leq q\leq 80\\ \frac{1}{\left\{1+{\left[0.3\left(q-80\right)\right]}^{-2}\right\}}, & 80<q<90\\ \frac{x}{100}, & 90\leq q\leq 100 \end{array}$$

(b).*U*_*qJ*_(*q*)

Considering the decreasing half of the Cauchy distribution

(23) $$U_{qJ}\left(q\right)=\{\begin{array}{ll} 1, & q\leq a\\ \frac{1}{\left\{1+{\left[\alpha \left(q-a\right)\right]}^{\beta }\right\}}, & q>a \end{array}$$

Specifically, α, β>0. If *a* = 55, β = 2, then *U*_*qJ*_(60) = 0.5, yielding α = 0.2.

(24) $$U_{qJ}\left(q\right)=\{\begin{array}{ll} 1, & q\leq 55\\ \frac{1}{\left\{1+{\left(\frac{q-55}{5}\right)}^{2}\right\}}, & 55\leq q\leq 100 \end{array}$$

(c).*U*_*qI*_(*q*)

Considering the Cauchy distribution directly, *a* = 75, α = 1/7.5. The membership function is calculated as follows:

(25) $$U_{qI(q)=\frac{1}{\left\{1+{\left(\frac{q-75}{7.5}\right)}^{2}\right\}}}$$

When assessing the matching degree of R peak detection, we calculate the value of qSQI according to Equations (22), (24), and (25). We can obtain qSQI single factor evaluation results: *r*_1_ = (*r*_11_, *r*_12_, *r*_13_).

*Single factor evaluation of pSQI*. This evaluation is similar to the structure of qSQI. According to the power spectrum distribution of pSQI's identification criteria (4), its performance level and its corresponding evaluation object value exhibit a trapezoidal distribution. Therefore, the trapezoidal distribution function is adopted as the membership function of pSQI. Assuming that p is the value of the power spectrum distribution, *p*∈[0, 1], we obtain the membership function of pSQI quality level *U*_*pH*_(*p*), *U*_*pI*_(*p*), and *U*_*pJ*_(*p*):

(26) $$U_{pH}\left(p\right)=\{\begin{array}{ll} 0, & x\leq 0.25\\ 0.1\left(x-0.25\right), & 0.25<x<0.35\\ 1, & x\geq 0.35 \end{array}$$

(27) $$U_{pJ}\left(p\right)=\{\begin{array}{ll} 1, & x<0.15\\ 0.1\left(0.25-x\right), & 0.15\leq x\leq 0.25\\ 0, & x>0.25 \end{array}$$

(28) $$U_{pI}\left(p\right)=\{\begin{array}{ll} 0, & x<0.18\\ 25\left(x-0.18\right), & 0.18\leq x<0.22\\ 1, & 0.22\leq x<0.28\\ 25\left(0.32-x\right), & 0.28\leq x<0.32\\ 0, & x\geq 0.32 \end{array}$$

We calculate the value of pSQI according to Equations (26)–(28), and pSQI's single factor evaluation results can be obtained: *r*_2_ = (*r*_21_, *r*_22_, *r*_23_).

*Single factor evaluation of kSQI*. According to the identification criteria (10) of Kurtosis kSQI, its performance level and its corresponding evaluation object value exhibits a rectangular distribution. Accordingly, we choose the rectangular distribution function to be the membership function of kSQI. We calculate the value of kSQI, and the result of single factor evaluation *r*_3_ = (*r*_31_, *r*_32_, *r*_33_) is obtained as follows:

(29) $$\{\begin{array}{ll} if\;kSQI>5, & r_{3}=(1,0,0)\\ if\;kSQI\leq 5, & r_{3}=(0,0,1) \end{array}$$

*Single factor evaluation of basSQI*. This evaluation is the same as that reported for qSQI. For the analysis of Equation (12), the baseline relative powers performance level and corresponding evaluation object value approximate a Cauchy distribution. We select the Cauchy distribution function to construct *U*_*bH*_(*b*), *U*_*bI*_(*b*), and *U*_*bJ*_(*b*). We calculate the value of basSQI according to Equations (30)–(32), and basSQI's single factor evaluation results can be obtained: *r*_4_ = (*r*_41_, *r*_42_, *r*_43_).

(30) $$U_{bH}\left(b\right)=\{\begin{array}{ll} 0, & 0\leq b\leq 90\\ \frac{1}{\left\{1+{\left[0.8718\left(b-90\right)\right]}^{-2}\right\}}, & 90<q<95\\ \frac{x}{100}, & 95\leq q\leq 100 \end{array}$$

(31) $$U_{bJ}\left(b\right)=\{\begin{array}{ll} 1, & q\leq 85\\ \frac{1}{\left\{1+{\left(\frac{b-85}{5}\right)}^{2}\right\}}, & 85<q\leq 100 \end{array}$$

(32) $$U_{bI}\left(b\right)=\frac{1}{\left\{1+{\left(\frac{b-92}{2.5}\right)}^{2}\right\}}$$

After repeating the single factor evaluation four times, we obtain the fuzzy matrix $R={\left[r_{1}\;r_{2}\;r_{3}\;r_{4}\right]}^{T}$.

##### Determine the weight vector W

**Figure 7:**
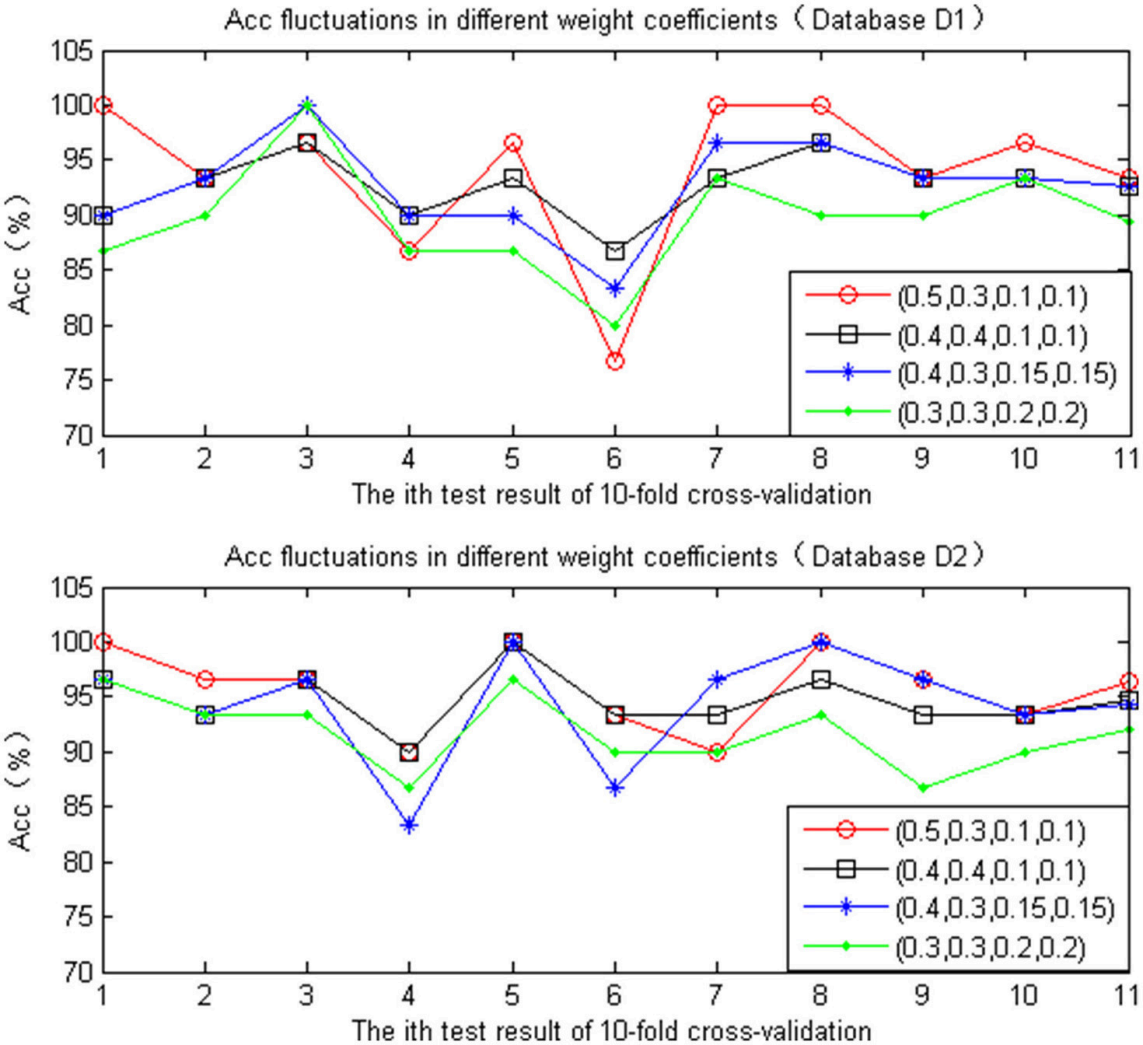
Accuracy comparison chart of different weight coefficient.

Different evaluation factors have different effects on the quality of ECG signals. Therefore, the selection of weight coefficients will have a great influence on the final quality assessment results. In this paper, different sets of weight vectors are selected to compare the four factors, which are verified according to 10 replicates of the 10-fold cross-validation test in the Database D1 and D2. The statistic is presented in Figure 7. Under different weight values of different SQI decision values, the accuracy of the quality of the assessment of ECG also differs. When the ratios of the four were set as follows (0.4, 0.4, 0.1, 0.1), whether the database D1 or D2, the accuracy of the ECG quality assessment under different test set was relatively high, with minimal fluctuation.

##### Determine the fuzzy operator

The principle of fuzzy comprehensive evaluation is fuzzy transformation. Numerous types of operation modes of fuzzy transformation are available. The commonly used fuzzy synthesis operators are classified as the following types:

1. *M*(∧, ∨) operator: Main factor determinant
2. *M*(·, ∨) operator: Main factor protruding
3. *M* (∧, ⊕) operator: Unbalance mean
4. *M* (·, ⊕) operator: Weighted mean

Considering the role of reflection, the first step is more appropriate for a multiplicative operation. In contrast, from a comprehensive point of view, it is appropriate to use the “boundedness and” operation to ensure the full use of all aspects of the information provided by the fuzzy vector R. In this paper, we need to synthesize the four SQIs' indicators to evaluate the effect of ECG quality, so we use the operator *M* (·, ⊕), which is also known as a bounded operator.

##### Determine the decision-making methods

After fuzzy synthesis, the vector *S* = (s_1_, s_2_, s_3_) of the fuzzy comprehensive evaluation is obtained, which provides abundant information. In this paper, we need to weigh the four single-factor evaluations for each tester to obtain the numerical result of rating class V. Therefore, further processing is needed. Commonly used methods include the principle of maximum membership degree, the principle of weighted membership degree, and the fuzzy vector single-value method. We choose the principle of weighted average decision-making, which is expressed as follows:

(33) $$V=\frac{\sum_{j\;=\;1}^{3}s_{j}{}^{2}\cdot j}{\sum_{j\;=\;1}^{3}s_{j}{}^{2}}$$

After the above four-step parameter setting, we establish the SQI quality evaluation mechanism based on fuzzy comprehensive evaluation and obtain the final evaluation rating set V as follows:

(34) $$ECG\{`\begin{array}{l} Excellent\;(E),v\leq 1.50;\\ Barely\;acceptable\;(B),1.50<v<2.40;\\ Unacceptable\;(U),v\geq 2.40. \end{array}$$

**Figure 8:**
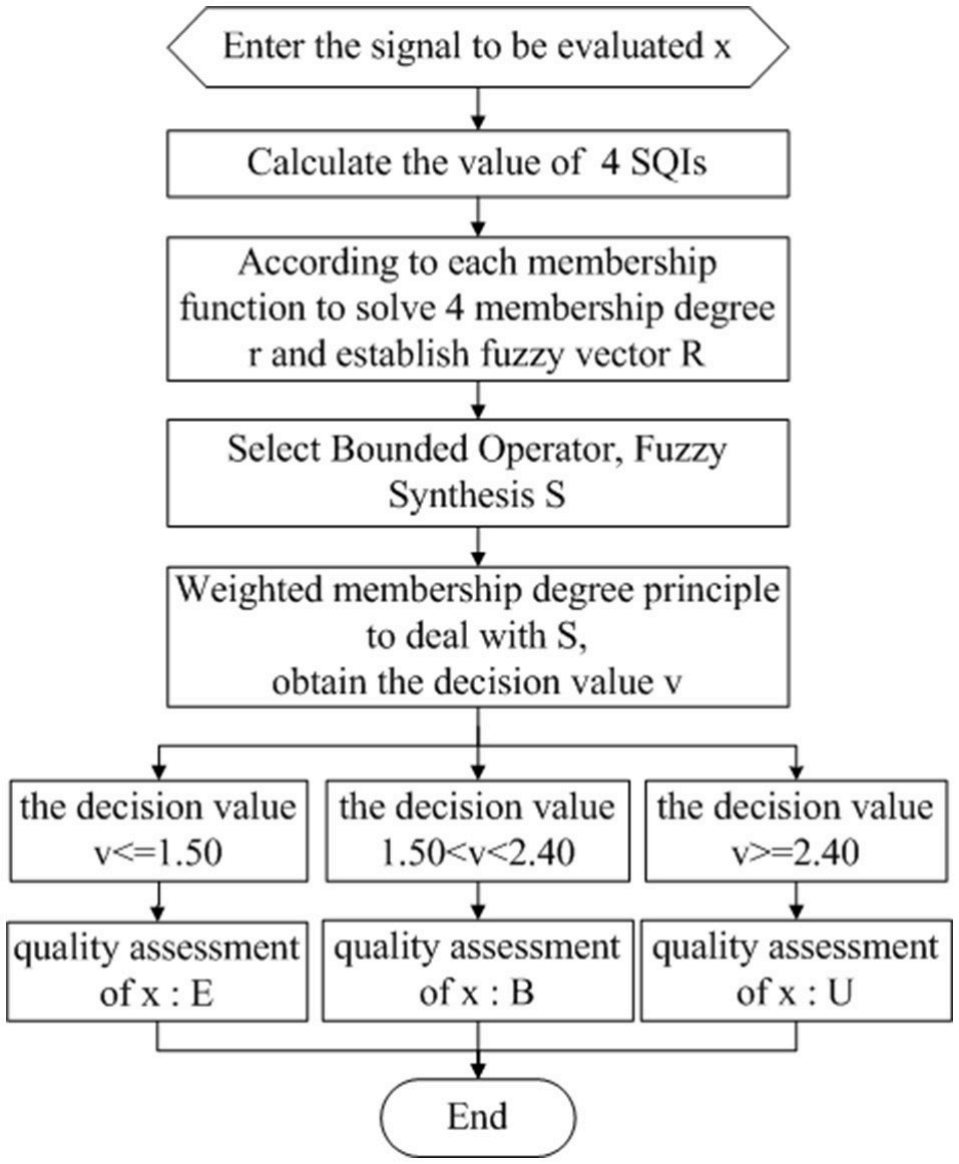
ECG quality assessment flow chart.

Then, for any ECG signal to be evaluated, the SQI quality evaluation mechanism based on fuzzy comprehensive evaluation can be used to obtain the ECG quality assessment results (shown in Figure 8) according to the above formula (33).

After the ECG quality assessment, the results were analyzed:

1. If E, the ECG signal quality is good. The signal can be directly entered into the identification, security monitoring or other applications.
2. If U, analyze the 4 SQIs: If kSQI or basSQI are unqualified, noise artifacts are present. Perform de-noising first, and then reevaluate the ECG quality. If pSQI or qSQI are unqualified, recollect the tester's ECG.
3. If B, ECG quality assessment should be performed again. If the result is E, the signal is treated as in method one. Otherwise, treated as in method two.

## Results and discussion

The results of the comparison of Fuzzy comprehensive evaluation with the simple heuristic fusion of the SQIs with Database D test are shown in Tables 4, 5 below:

**Table 4:** Performances of simple heuristic fusion of the SQIs and fuzzy comprehensive evaluation.

**Method**	**SQI entered**	**Training performance (%)**			**Test performance (%)**		
		**Acc**	**Se**	**Sp**	**Acc**	**Se**	**Sp**
Simple heuristic fusion	4	85.67	94.67	88.67	84.33	88.67	81.33
Simple heuristic fusion	5	88.67	87.33	85.33	87.00	88.67	87.33
Fuzzy comprehensive evaluation	4	89.33	93.67	74.33	**92.67**	**97.33**	**88.67**

*The best performing algorithm (on the independent Database D1) is indicated in bold*.

**Table 5:** Performances of simple heuristic fusion of the SQIs and fuzzy comprehensive evaluation.

**Method**	**SQI entered**	**Training performance (%)**			**Test performance (%)**		
		**Acc**	**Se**	**Sp**	**Acc**	**Se**	**Sp**
Simple heuristic fusion	4	92.00	94.67	92.00	91.33	93.67	92.67
Simple heuristic fusion	5	91.67	92.33	89.67	89.67	91.00	88.33
Fuzzy comprehensive evaluation	4	97.67	96.33	98.33	**94.67**	**90.33**	**93.00**

*The best performing algorithm (on the independent database D2) is indicated in bold*.

For simple heuristic fusion of the SQIs, when the number of SQIs increases from 4 to 5, the accuracy of the database D1 and D2 is not well optimized, even in its sensitivity (Se) and specificity (Sp). These values are not increasing but are decreasing. These findings indicate that the new evaluation parameter cSQI contains information that is complementary to the original (qSQI, pSQI, kSQI, basSQI), which affects the evaluation of ECG quality. Therefore, the selection (qSQI, pSQI, kSQI, basSQI) is more reasonable. Compared with simple heuristic fusion of the SQIs, although the same number of SQI indicators is used to quantify the different characteristics of ECG signals, the accuracy of database D1 and D2 is improved after it is synthesized by fuzzy comprehensive evaluation.

**Figure 9:**
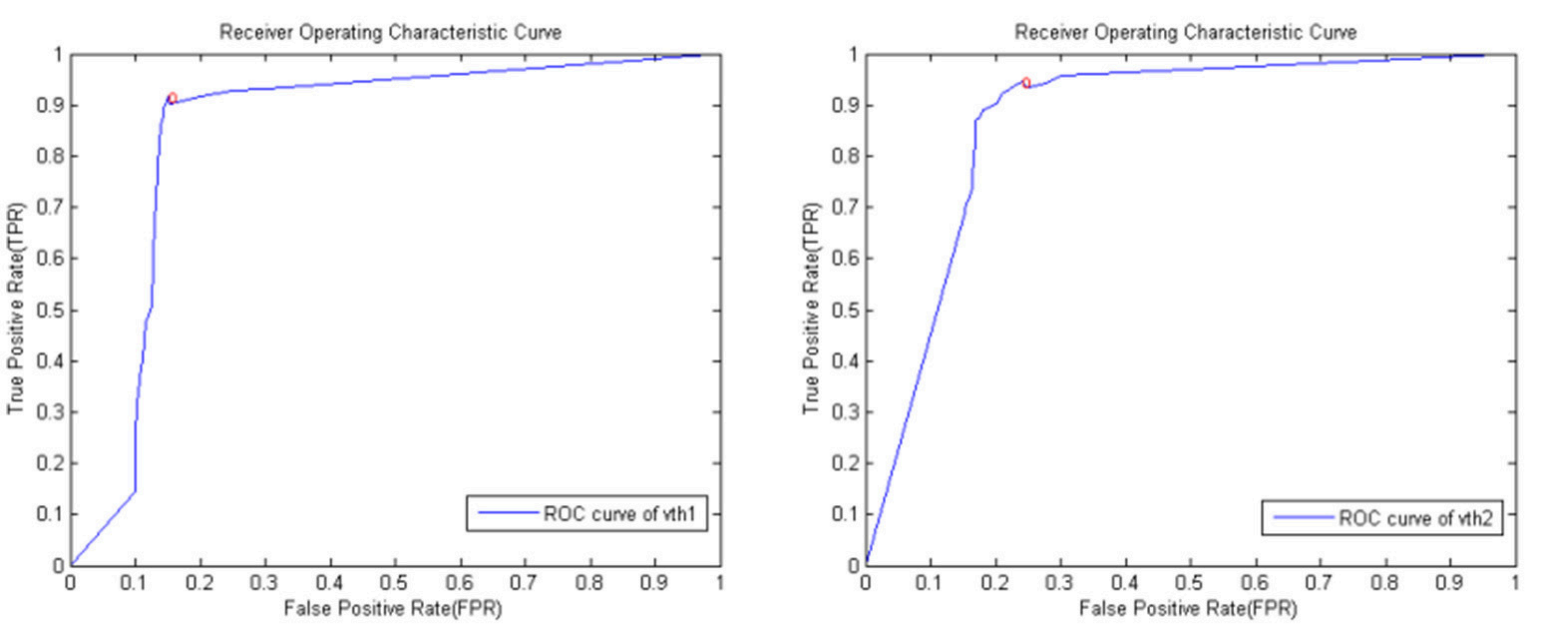
ROC curve derived by varying v across the database D2. The circle indicates the position of maximum accuracy (91.67% with *v*_*th*1_, 94.67% with *v*_*th*1_ and *v*_*th*2_).

Simultaneously, the above Tables 4, 5 shows that the database D2 shows a better accuracy than D1. Using the database D2, we varied the value of v above for which data were considered to be excellent (E) or barely acceptable (B) quality and calculated the receiver operating characteristic (ROC) curve (Figure 9). The value v that yielded the best classification accuracy was *v*_*th*1_ = 1.50, *v*_*th*2_ = 2.40 (in the ROC curve, they were normalized to *v*_*th*1_ = 0.15, *v*_*th*2_ = 0.24), which resulted in an accuracy of 91.67% (275 correctly classified out of 300) on the database D2, using the threshold *v*_*th*1_. Then adding the threshold *v*_*th*2_, the accuracy on the test set was found to be 94.67% (284 correctly classified out of 300).

To perform a more extensive and accurate comparative performance evaluation, the base performance of the proposed system is compared with the four existing algorithms, (Johannesen, 2011; Clifford et al., 2012; Martínez-Tabares et al., 2012; Shahriari et al., 2017), all of which adopted the Database D2, used single-lead ECG signal, made the comparison more persuasive. The experimental results are presented in Table 6.

**Table 6:** Contrast tabulation of experimental results for different quality evaluation algorithms.

**Authors**	**Methods**	**Performance of Database D2(%)**		
		**Acc**	**Se**	**Sp**
G D Clifford (Clifford et al., 2012)^1, 2^	SVM+SQIs	**97.80**	**96.30**	**99.30**
FJ Martínez-Tabares (Martínez-Tabares et al., 2012)^4^	Diversity systems	**96.00**	86.00	91.00
Yalda Shahriari (Shahriari et al., 2017)^5^	SSIM	93.10	**96.30**	90.00
Lars Johannesen (Johannesen, 2011)^1, 3^	SQIs	88.00		
Fuzzy Comprehensive Evaluation + SQIs		**94.67**	**90.33**	**93.00**

*Best results are highlighted. The results of this article are underlined*.

1. Based on SQI indexes.
2. Four SQI indexes were extracted and fused by support vector machine (SVM) and multi-layer perceptron (MLP), which achieved high accuracy. However, the calculation of the index bSQI (the percentage of beats detected by *wqrs* that were also detected by *eplimited*) is pretty complicated.
3. The author adopted five SQI indexes, then considered each index in turn, at each step in the algorithm ECGs are grouped into two groups depending on a set of ECG features (SQI), but the accuracy rate is poor.

Compared with these two studies, we propose the SQI Quality Evaluation Mechanism Based on Fuzzy Comprehensive Evaluation, with only 4 SQI indexes, all of which are simply calculated, and obtain a good accuracy.

4. A Correlation and Diversity-based Approaches is proposed.
5. Adopt a Structural Similarity Measure (SSIM) to compare images of two ECG records that are obtained from displaying ECGs in a standard scale.

Compared with these two algorithms, we use the SQI index to quantify the quality of ECG which increased the readability of this algorithm, made it easy to be understood, and obtains an ideal accuracy. Therefore, the algorithm proposed in this paper has some advantages compared with other algorithms reported in the literature.

## Conclusion

We have described an effective system (with an accuracy of 92.67% on database D1 and 94.67% on database D2) that could be deployed as a stand-alone signal quality assessment algorithm to vet the clinical utility of ECG signals. Applications range from determining the quality of ECG signal collected to false alarm suppression. Moreover, the algorithm presented here is quite general and could be retrained and applied to any periodic or quasi-periodic signal, such as contraction signals.

Future work should focus on methods for expanding the feature space and on the further optimization of feature fusion.

## Author contributions

ZZ and YZ developed the concept of this review and wrote the manuscript.

### Conflict of interest statement

The authors declare that the research was conducted in the absence of any commercial or financial relationships that could be construed as a potential conflict of interest.
